# Generation and characterization of two human induced pluripotent stem cell (hiPSC) lines homozygous for the Apolipoprotein e4 (APOE4) risk variant—Alzheimer's disease (ASUi005-A) and healthy non-demented control (ASUI006-A)

**DOI:** 10.1016/j.scr.2018.09.007

**Published:** 2018-09-21

**Authors:** Nicholas Brookhouser, Ping Zhang, Richard Caselli, Jean J. Kim, David A. Brafman

**Affiliations:** a School of Biological and Health Systems Engineering, Arizona State University, Tempe, AZ 85287, USA; b Molecular and Cellular Biology, Stem Cells and Regenerative Medicine Center, Baylor College of Medicine, Houston, TX 77030, USA; c Department of Neurology, Mayo Clinic College of Medicine, Scottsdale, AZ 85259, USA

## Abstract

Although the majority of late-onset Alzheimer's disease (AD) patients are labeled sporadic, multiple genetic risk variants have been identified, the most powerful and prevalent of which is the e4 variant of the Apolipoprotein E (APOE) gene. Here, we generated human induced pluripotent stem cell (hiPSC) lines from the peripheral blood mononuclear cells (PBMCs) of a clinically diagnosed AD patient [ASUi005-A] and a non-demented control (NDC) patient [ASUi006-A] homozygous for the APOE4 risk allele. These hiPSCs maintained their original genotype, expressed pluripotency markers, exhibited a normal karyotype, and retained the ability to differentiate into cells representative of the three germ layers.

**Resource table:** 

Unique stem cell lines identifier	ASUi005-A ASUi006-A
Alternative names of stem cell lines	ASU-161 ASU-487
Institution	Arizona State University; Tempe, AZ; USA
Contact information of distributor	David Brafman, David.Brafman@asu.edu
Type of cell lines	iPSC
Origin	Human
Cell source	Human peripheral blood mononuclear cells (PBMCs)
Clonality	Clonal
Method of reprogramming	CytoTune^®^-iPS 2.0 Reprogramming System
Multiline rationale	Age-matched Alzheimer's disease and non-demented control hiPSC lines homozygous for the APOE 4 risk factor
Gene modification	No
Type of modification	N/A
Associated disease	Alzheimer's disease
Gene/locus	Apolipoprotein E (APOE)
Method of modification	N/A
Name of transgene or resistance	N/A
Inducible/constitutive system	N/A
Date archived/stock date	March 2018
Cell line repository/bank	Not applicable
Ethical approval	Mayo Clinic Institutional Review Board; IRB # 15-008674

## Resource utility

Polymorphisms in the Apolipoprotein (APOE) gene have been identified as the most prevalent of the risk factors associated with sporadic Alzheimer's disease (AD). As such, hiPSCs with various APOE genotypes will provide a valuable resource to study the mechanisms by which this risk factor contributes to AD onset and progression.

## Resource details

Genome-wide association studies (GWAS) studies have identified several risk factors associated with increased probability of sporadic Alzheimer's disease (SAD) onset (Bettens et al., 2010). Of these risk factors, polymorphism in the Apolipoprotein E (APOE) gene, a lipoprotein transporter involved in cholesterol metabolism, is the strongest and most prevalent (Hauser & Ryan, 2013). Compared to individuals with an APOE e3/3 genotype (referred to as the ‘risk neutral’ allele), heterozygosity for the e4 allele increases AD risk by 3 fold, and homozygosity for the e4 allele increases risk up to 12 fold (Wolf et al., 2013). In this study, we report the generation of hiPSCs from two individuals from the Arizona APOE Cohort (for which recruitment and enrolment strategies have been described previously (Caselli et al., 2011)) that are homozygous for the APOE e4 allele— a clinically diagnosed AD patient (ASUi005-A, Mini-Mental Status Exam [MMSE] score = Patient too advanced to collect data.) who fulfilled published diagnostic criteria (McKhann et al., 2011) and an age-matched non-demented control patient (NDC; ASUi006-A, MMSE score = 29) (Table 1).

**Fig. 1.**
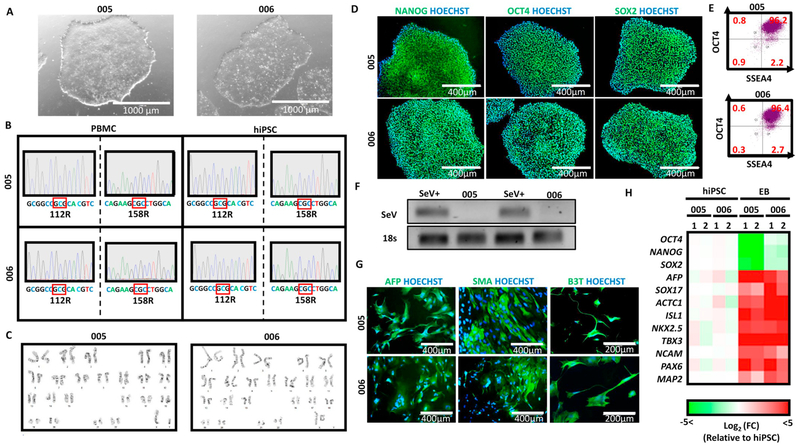
(A) Phase contrast images of hiPSC lines. (B) Sanger sequencing showing maintenance of genotype at ApoE locus. (C) Karyotyping confirmed cells maintained a normal euploid karyotype. (D-E) Immunofluorescent and flow cytometry analysis of pluripotency markers (NANOG, OCT4, SOX2, SSEA4). (F) RT-PCR demonstrates absence of SeV vector. SeV infected fibroblasts were used as a positive control. (G) Immunofluorescent staining and (H) gene expression analysis of in vitro differentiated cells shows expression of markers associated with endoderm (AFP, SOX17), mesoderm (ACTC1, ISL1, SMA, TBX3), and ectoderm (B3T, MAP2, NCAM, PAX6).

Peripheral blood mononuclear cells (PBMCs) were reprogrammed into hiPSCs using the non-integrating CytoTune^®^-iPS 2.0 Reprogramming System (Thermo Fisher Scientific). Several clones from each patient were isolated, expanded, and characterized by karyotyping and flow cytometry. One clone was expanded and fully characterized for each line (Fig. 1 and Table 2). The expanded hiPSC clones displayed a typical pluripotent stem cell morphology (Fig. 1A). All expanded clones were confirmed to be negative for mycoplasma (Supplementary Table 1). Sequencing analysis of the hiPSCs at the APOE gene in exon 4 confirm homozygosity at the e4 allele, identical to the parental PBMCs [Fig. 1B; Note: Human APOE has three major isoforms, ApoE2, ApoE3, and ApoE4, which differ by two amino acid substitutions at residues 112 and 158 in exon 4—ApoE2 (Cys112, Cys158), ApoE3 (Cys112, Arg158), ApoE4 (Arg112, Arg158)]. Expanded clones maintained a normal euploid karyotype (Fig. 1C). Immunofluorescent staining (Fig. 1D) and flow cytometry (Fig. 1E) revealed that the hiPSCs expressed high levels of pluripotency-associated markers NANOG, OCT4, SOX2, and SSEA-4. Absence of viral transgenes in expanded clones was confirmed by RT-PCR (Fig. 1F). Finally, to verify pluripotency, hiPSCs were spontaneously differentiated in vitro through embryoid body (EB) formation. Immunofluorescence (Fig. 1G) and gene expression analysis (Fig. 1H) of EBs revealed downregulation of pluripotency-associated markers (OCT4, NANOG, SOX2) and upregulation of genes associated with endoderm (AFP, SOX17), mesoderm (ACTC1, ISL1, SMA, TBX3), and ectoderm (B3T, MAP2, NCAM, PAX6).

## Materials and methods

### Reprogramming of PBMCs

Peripheral blood samples were collected in BD Vacutainer cell preparation tubes and centrifuged for 30 min at 1800 RCF. Isolated PBMCs were cultured in expansion medium (EM; QBSF-60 [Fisher Scientific] supplemented with 100 μg/mL Primocin [Fisher Scientific], 1% penicillin/streptomycin [Thermo Fisher], 50 μg/mL ascorbic acid [Sigma], 50 ng/mL SCF [R&D], 10 ng/mL IL-3 [R&D], 2 U/mL EPO [R& D], 40 ng/mL IGF-1 [R&D], 1 μM Dexamethasone [Sigma]). After 9–12 days of expansion, 2.5 × 10^5^ PBMCs were resuspended in EM and transferred to a 12 well plate. Sendai viruses (SeV; CytoTune^®^-iPS 2.0 Reprogramming Kit [Thermo Fisher]) were added at a multiplicity of infection MOI of 10:10:6 for the hKOS:c-Myc:Klf4 Sendai viruses. Three days after transduction, cells were cultured on hESC-qualified Matrigel^®^ (Corning) in TeSR-E7 medium for 7 days, and then switched to TeSR-E8 (E8) medium (STEMCELL Technologies). After 21 days, individual hiPSC colonies were mechanically isolated and expanded in a 37 °C incubator with 5% CO_2_. After mechanically passaging for the first 3 passages, hiPSCs were non-enzymatically passaged using ReLeSR™ (STEMCELL Technologies) at a split ratio of 1:4–1:6 and cryopreserved. For routine passaging of these lines, Versene was used at a split ratio of 1:6 with 5 μM Rho kinase inhibitor (Y-27632; Biogems). Mycoplasma testing was performed with the MycoAlert PLUS kit (Lonza) and the Lucetta™ Luminometer (Lonza).

### Quantitative RT-PCR (QPCR)

RNA was isolated from cells (NucleoSpin RNA Kit, Clontech) and reverse transcription was performed (iScript RT Supermix, Bio-Rad). QPCR was carried out using SYBR green dye on a CFX384 Touch™ Real-Time PCR Detection System. QPCR experiments run with SYBR green dye were carried out using iTaq Universal SYBR Green Supermix (Bio-Rad). For qPCR experiments run with SYBR green dye, a 2 min gradient to 95 °C followed by 40 cycles at 95 °C for 5 s and 60 °C for 30 s was used. Primer sequences are provided in Table 3. Gene expression was normalized to 18S rRNA levels. Relative fold changes in gene expression were calculated using the 2 – AACt method.

### SeV detection

After a minimum of 10 passages, RNA was isolated from cells (NucleoSpin RNA Kit, Clontech) and reverse transcription was performed (iScript RT Supermix, Bio-Rad). RT-PCR was run on a Bio-Rad CFX384 Real-Time System with the primers listed in Table 3 and the following cycling parameters—a 2 min gradient to 95 °C followed by 20 cycles at 95 °C for 5 s and 60 °C for 30 s. The resulting products were then run on a 1% gel.

### APOE genotyping

For APOE genotyping via Sanger sequencing, genomic DNA was isolated from cells using the DNeasy kit (Qiagen). PCR was performed on a MultiGene OptiMax thermal cycler with the primers list in Table 3 and the following cycling parameters—30 s at 98 °C followed by 35 cycles at 95 °C for 15 s, 62 °C for 30 s, and 72 °C for 30 s with a final extension of 10 min at 72 °C. The resulting PCR product was cleaned up using the PureLink™ PCR Purification Kit (ThermoFisher). Sanger sequencing was performed on PCR products (ASU CLAS Genomics Facility) uses Big Dye V3.1 chemistry with samples processed using an Applied Biosystems 3730XL Sequence Analysis Instrument.

### Karyotyping and STR analysis

Cytogenetic analysis was performed using standard protocols for G-banding (Baylor Miraca Genetics Laboratories). For ASUi005-A, cells were tested at passage 10, 20 metaphase cells were counted, and 4 cells were karyotyped. For ASUi006-A, cells were tested at passage 8, 20 metaphase cells were counted, and 3 cells were karyotyped. Short tandem repeat (STR) analysis was performed with Promega's PowerPlex^®^ 16 multiplex STR system (Cell Line Genetics). The following loci were tested: Amelogenin, CSF1PO, D13S317, D16S539, D18S51, D21S11, D3S1358, D5S818, D7S820, D8S1179, FGA, Penta D, Penta E, THO1, TPOX, vWA.

### Flow cytometry

Cells were dissociated with Accutase for 10 min at 37 °C, triturated, and passed through a 40 μm cell strainer. Cells were then washed twice with stain buffer (BD Biosciences) and resuspended at a maximum concentration of 5 × 10^6^ cells per 100 μL. Cells were fixed for 30 min at RT with BD Cytofix Fixation Buffer (BD Biosciences). The cells were then washed twice with stain buffer and permeabilized with BD Phosflow Perm Buffer III (BD Biosciences) for 30 min on ice. Cells were then washed twice with stain buffer. Antibodies were added at the dilution indicated in Table 3 in 100 μL of cell suspension. Cells were stained with primary antibodies for 1 h on ice, washed, and resuspended in stain buffer. Cells were analyzed by an LSR II flow cytometer (BD Biosciences). Gates were determined using isotype only controls.

### Immunofluorescence

Cells were gently washed twice with PBS prior to fixation. Cells were then fixed for 20 min at room temperature (RT) with BD Cytofix Fixation Buffer (BD Biosciences). Cells were then washed twice with PBS and permeabilized with BD Phosflow Perm Buffer III (BD Biosciences) for 30 min at 4OC. Cells were then washed twice with PBS. Primary antibodies were incubated overnight at 4°C and then washed twice with PBS at RT. Secondary antibodies were incubated at RT for 1 h. Antibodies and the concentrations that were used are listed in Table 3. Nucleic acids were stained for DNA with Hoechst 33342 (2 μg/mL; ThermoFisher) for 10 min at RT and then washed twice with PBS. Imaging was performed using an EVOS FL Cell Imaging System (ThermoFisher).

### In vitro embryoid body (EB) formation

HiPSCs were harvested using ReLeSR™ (StemCell Technologies) and plated on low attachment plates in E8 medium. The following day, the media was changed to differentiation medium (DM; DMEM/F12, 20% FBS, 1% Pen/Strep). After 5 days, EBs were plated on Matrigel-coated plates and cultured with DM. After 14 days, cells were fixed, permeabilized, and stained for germ layer markers. In addition, cells were dissociated using Accutase, RNA was isolated (as described above for qPCR), and qPCR was performed (as described above for qPCR) to assess expression of pluripotency and germ layer markers.

## Supplementary Material

1

## Figures and Tables

**Table 1:** Summary of lines.

iPSC line names	Abbreviation in figures	Gender	Age	Ethnicity	Genotype of locus	Disease
ASUi005-A	005	M	87	N/A	APOE: 112R/158R	Alzheimer's disease
ASUi006-A	006	F	86	N/A	APOE: 112R/158R	Healthy/Non-Demented

**Table 2:** Characterization and validation.

Classification	Test	Result	Data
Morphology	Photography	Normal	Fig. 1 A
Phenotype	Qualitative analysis: Immunocytochemistry	Positive staining for OCT4, NANOG, and SOX2	Fig. 1D
	Quantitative analysis: Flow cytometry	OCT4/SSEA-4 Double Positive > 95%	Fig. 1E
Genotype	Karyotype (G-banding) and resolution	46XY (ASUi005-A) 46XX (ASUi006-A) Resolution 450–550	Fig. 1C
Identity	Microsatellite PCR (mPCR)	Not performed	
	STR analysis	16 Loci All matched	Available with the authors
Mutation analysis	Sequencing Southern Blot OR WGS	Homozygous for Apolipoprotein e4 risk variant Not performed	Fig. 1B
Microbiology and virology	Mycoplasma	Mycoplasma testing by luminescence. Negative	Supplementary Table 1
Differentiation potential	Embryoid body	Endoderm (AFP, SOX17), mesoderm (ACTC1, ISL1, NKX2.5, TBX3, SMA), and ectoderm (B3T, NCAM, PAX6, MAP2)	Fig. 1G and H
Donor screening (OPTIONAL)	HIV 1+2 Hepatitis B, Hepatitis C	Not performed	
Genotype additional info	Blood group genotyping	Not performed	
(OPTIONAL)	HLA tissue typing	Not performed	

**Table 3:** Reagent details.

Antibodies used for immunocytochemistry and flow cytometry			
	Antibody	Dilution	Company Cat # and RRID
Pluripotency markers	Mouse anti-OCT4	1:50	Santa Cruz, Cat# sc-5279 RRID: 628051
	Mouse anti-NANOG	1:50	Santa Cruz, Cat# sc-293121 RRID: 10548762
	Goat anti-SOX2	1:50	Santa Cruz, Cat# sc-17320 RRID: 2286684
	Mouse IgG1 anti-OCT4-PE	1:10	BD Biosciences, Cat# 560186 RRID: 1645331
	Mouse IgG3 anti-SSEA4-APC	1:10	R&D Systems, Cat# FAB1435A RRID: 494994
Differentiation markers	Rabbit anti-AFP	1:50	Santa Cruz, Cat# sc-15375 RRID: 2223935
	Mouse-anti SMA	1:50	Santa Cruz, Cat# sc-53015 RRID: 628683
	Mouse anti-B3T	1:100	Fitzgerald, Cat# 10R-T136A RRID: 1289248
Secondary antibodies	Alexa 488 Donkey anti-goat IgG	1:200	Thermo Fischer, Cat# A11055 RRID: 2534102
	Alexa 647 Donkey anti-mouse IgG	1:200	Thermo Fisher, Cat# A31571 RRID: 162542
	Alexa 488 Donkey anti-rabbit IgG	1:200	Thermo Fisher, Cat# A21206 RRID: 141708
Isotype control	Mouse IgG1-PE	1:10	BD Biosciences, Cat# 559320 RRID: 397218
	Mouse IgG1-APC	1:10	R&D Systems, Cat# IC007A RRID: 952035

Primers			
	Target	Product size (bp)	Forward/Reverse primer (5′-30′
SeV Transgene (RT-PCR)	*SeV*	181	GGATCACTAGGTGATATCGAGC
			ACCAGACAAGAGTTTAAGAGATATGTATC
Pluripotency markers (qPCR)	*NANOG*	183	CAATGGTGTGACGCAGGGAT
			GGACTGTTCCAGGCCTGATT
	*OCT4*	164	CAAAGCAGAAACCCTCGTGC
			CTCGGACCACATCCTTCTCG
	*SOX2*	123	GGATAAGTACACGCTGCCCG
			ATGTGCGCGTAACTGTCCAT
Germ layer markers (qPCR)	*ACTC1*	179	GTACCCTGGTATTGCTGATCG
			CCTCATCGTACTCTTGCTTGCT
	*AFP*	301	AGAGTTGCTAAAGGATACCAGGA
			AGGCCAATAGTTTGTCCTCAC
	*ISL1*	135	GGATTTGGAATGGCATGCGG
			CATTTGATCCCGTACAACCTGA
	*MAP2*	111	CCAGTTTCTGCGCCCAGATTT
			AGCTCCCAATCAATGCTTCCT
	*NCAM*	77	AGACCCCATTCCCTCCATCA
			TGTGCCCATCCAGAGTCTTT
	*NKX2.5*	149	GAGCCGAAAAGAAAGCCTGAA
			CACCGACACGTCTCACTCAG
	*SOX17*	100	GAATCCAGACCTGCACAACG
			CTCTGCCTCCTCCACGAAG
	*TBX3*	212	ATTTCACAATTCTCGGTGGA
			TATAATTCCCCTGCCACGTA
	*PAX6*	100	CTTCGCTAATGGGCCAGTGA
			TCAGATTCCTATGCTGATTGGTGA
Housekeeping gene (qPCR)	*18s*	150	GTAACCCGTTGAACCCCATT
			CCATCCAATCGGTAGTAGCG
Genotyping (PCR)	*APOE*	244	TAAGCTTGGCACGGCTGTCCAAGGA
			ACAGAATTGGCCCCGGCCTGGTACAC
