# Supplementary material for: Generation and characterization of two human induced pluripotent stem cell (hiPSC) lines homozygous for the Apolipoprotein e4 (APOE4) risk variant—Alzheimer's disease (ASUi005-A) and healthy non-demented control (ASUI006-A)
Source: Stem Cell Res. Author manuscript; Available in PMC 2018 Nov 5. (PMC6217860; doi:10.1016/j.scr.2018.09.007)
Supplement: 1 [file NIHMS1509780-supplement-1.pdf]

Supplementary Table 1. Mycoplasma testing of expanded hiPSC clones.

Sample	Reading A (RLU/s)	Reading B (RLU/s)	Ratio (B/A)	Result
**ASUi005-A**	13888	4503	0.3242	**Negative**
**ASUi006-A**	69455	21127	0.3041	**Negative**
Positive Control	5918	2278206	384.9621	Positive
Negative Control	8266	3797	0.4593	**Negative**
